# Clinicopathological characteristics and factors associated with axillary lymph node metastasis in multifocal/multicentric versus unifocal breast cancer: a retrospective study

**DOI:** 10.3389/fonc.2026.1864087

**Published:** 2026-09-10

**Authors:** Qiuchen Jiang, Juan Shen, Xinxin Wu, Wei Zhang

**Affiliations:** Department of Breast, Thyroid and Hernia Surgery, Liaocheng People’s Hospital, Liaocheng, Shandong, China

**Keywords:** axillary lymph node metastasis, firth logistic regression, lymphovascular invasion, multifocal/multicentric breast cancer, nomogram, unifocal breast cancer

## Abstract

**Background:**

Multifocal/multicentric breast cancer (MMBC) may exhibit a greater tumor burden and higher risk of lymph node involvement than unifocal breast cancer (UFBC). This study compared the clinicopathological characteristics and nodal positivity patterns of MMBC and UFBC and explored factors associated with axillary lymph node (ALN) metastasis.

**Methods:**

This retrospective single-center study included 66 patients with MMBC and 104 patients with UFBC treated between October 2017 and March 2020. Clinicopathological characteristics and sentinel lymph node and ALN positivity rates were compared between groups. Factors associated with ALN metastasis were evaluated separately in each group. Variables significant in univariate analyses were included in Firth-type bias-reduced logistic regression models. Exploratory nomograms were constructed, and model performance was assessed using the area under the receiver operating characteristic curve (AUC), Brier score, calibration analysis, and bootstrap internal validation.

**Results:**

Compared with UFBC, MMBC was associated with higher proportions of patients aged ≤50 years (53.03% vs. 36.54%; P = 0.034), lymphovascular invasion (LVI; 46.97% vs. 23.08%; P = 0.001), coexisting intraductal papilloma (13.64% vs. 1.92%; P = 0.002), and ALN positivity (40.9% vs. 20.2%; P = 0.003). Sentinel lymph node positivity did not differ significantly between groups (21.2% vs. 24.0%; P = 0.669). In UFBC, T2 stage (adjusted odds ratio [OR]=4.437, 95% confidence interval [CI]: 1.410-13.965), LVI (OR = 5.064, 95% CI: 1.614-15.891), and invasive micropapillary carcinoma (OR = 6.580, 95% CI: 1.331-32.523) remained significantly associated with ALN metastasis. In MMBC, ordinal T stage and LVI were significant in univariate analyses but not after mutual adjustment. The optimism-corrected AUCs were 0.746 for the UFBC model and 0.664 for the MMBC model; the corresponding optimism-corrected Brier scores were 0.142 and 0.232.

**Conclusions:**

In this exploratory retrospective study, MMBC was associated with higher frequencies of LVI, coexisting intraductal papilloma, and ALN positivity than UFBC. In UFBC, T stage, LVI, and IMPC remained significantly associated with ALN metastasis, whereas no factor remained statistically significant after mutual adjustment in MMBC. The UFBC model demonstrated moderate internally validated discrimination, whereas the MMBC model showed more limited performance. Given the small sample size, all findings and nomograms should be considered exploratory and require validation in larger independent cohorts.

## Introduction

Breast cancer is one of the most common malignancies among women worldwide (1). In 2020, approximately ^2.3^ million new cases and 685,000 deaths were reported globally, accounting for 24.5% of newly diagnosed cancers and 15.5% of cancer-related deaths among women (2). Its global incidence is projected to continue increasing over the coming decades (3).

Breast cancer can be classified according to pathological type, molecular characteristics, and the number and spatial distribution of tumor foci. Based on tumor distribution, breast cancer may be categorized as unifocal, multifocal, or multicentric. Multifocal breast cancer is generally defined as the presence of two or more malignant tumor foci within the same breast quadrant, separated by normal tissue, whereas multicentric breast cancer refers to multiple malignant foci located in different quadrants of the same breast (4). However, these definitions vary across studies, and some classifications include only invasive foci, whereas others also consider *in situ* lesions (5).

Although multifocal and multicentric breast cancers differ in their anatomical distribution and may not be biologically identical, they are frequently combined into a single multifocal/multicentric breast cancer (MMBC) category in retrospective clinicopathological studies. This approach is often adopted because of overlap in imaging and pathological classification, inconsistent historical definitions, and limited numbers available for reliable separate analyses. Nevertheless, findings based on a combined MMBC cohort should be interpreted with recognition of the potential biological and clinical heterogeneity between multifocal and multicentric disease.

Surrogate molecular subtype classification based on estrogen receptor, progesterone receptor, human epidermal growth factor receptor 2 (HER2), and Ki-67 expression is widely used in clinical research. However, recommendations regarding Ki-67 interpretation and surrogate subtype classification have evolved with updated international consensus guidelines (6, 7). In retrospective studies, the molecular-subtyping framework may therefore reflect the pathological criteria and institutional practices used during the original study period.

MMBC has been reported in approximately 10%-20% of patients with breast cancer, although its prevalence varies according to the definitions and diagnostic methods used (8). Previous studies have associated MMBC with greater overall tumor burden, higher frequencies of lymphovascular invasion and lymph node involvement, and increased risks of local recurrence or distant metastasis compared with unifocal breast cancer (UFBC) (9). Patients with MMBC are generally less likely to undergo conventional breast-conserving surgery and more likely to undergo mastectomy than patients with UFBC because of the distribution and extent of multiple tumor foci. Nevertheless, oncoplastic breast-conserving surgery may provide an appropriate surgical option for carefully selected patients with MMBC by facilitating wider tumor excision while preserving breast appearance (10). However, the prognostic significance of MMBC remains controversial after adjustment for tumor stage and other clinicopathological factors.

The assessment of tumor size in MMBC also remains challenging. Current staging systems determine T stage according to the diameter of the largest invasive focus, whereas the summed diameter of all invasive lesions may more fully describe the overall tumor burden (11). However, aggregate tumor size has not been standardized for clinical staging, and its additional clinical value remains uncertain. This distinction is particularly relevant when examining the relationship between tumor burden and axillary lymph node metastasis.

Axillary lymph node status is an important component of breast cancer staging and treatment planning (12). Although previous MMBC research has focused primarily on surgical management, inter-lesional heterogeneity, and molecular or genetic characteristics (13–18), the clinicopathological factors associated with axillary lymph node metastasis in MMBC remain insufficiently characterized. Moreover, sparse patient numbers in some pathological subgroups may complicate conventional regression analysis and require statistical methods that reduce sparse-data and separation-related bias (19, 20).

Therefore, further comparative investigation of MMBC and UFBC is needed to clarify their clinicopathological differences and identify factors associated with axillary lymph node metastasis. Such analyses should distinguish univariate associations from multivariable findings and interpret prediction models cautiously, particularly when sample sizes are limited.

### Objectives

This study aimed to compare the clinicopathological characteristics and nodal positivity patterns of patients with combined multifocal/multicentric breast cancer and unifocal breast cancer and to identify factors associated with axillary lymph node metastasis within each group. A secondary objective was to construct exploratory nomograms based on the corresponding Firth-type bias-reduced logistic regression models and to assess their discrimination, predictive accuracy, calibration, and internal validity.

## Materials and methods

### Study design and setting

#### Sample collection

**Figure 1:**
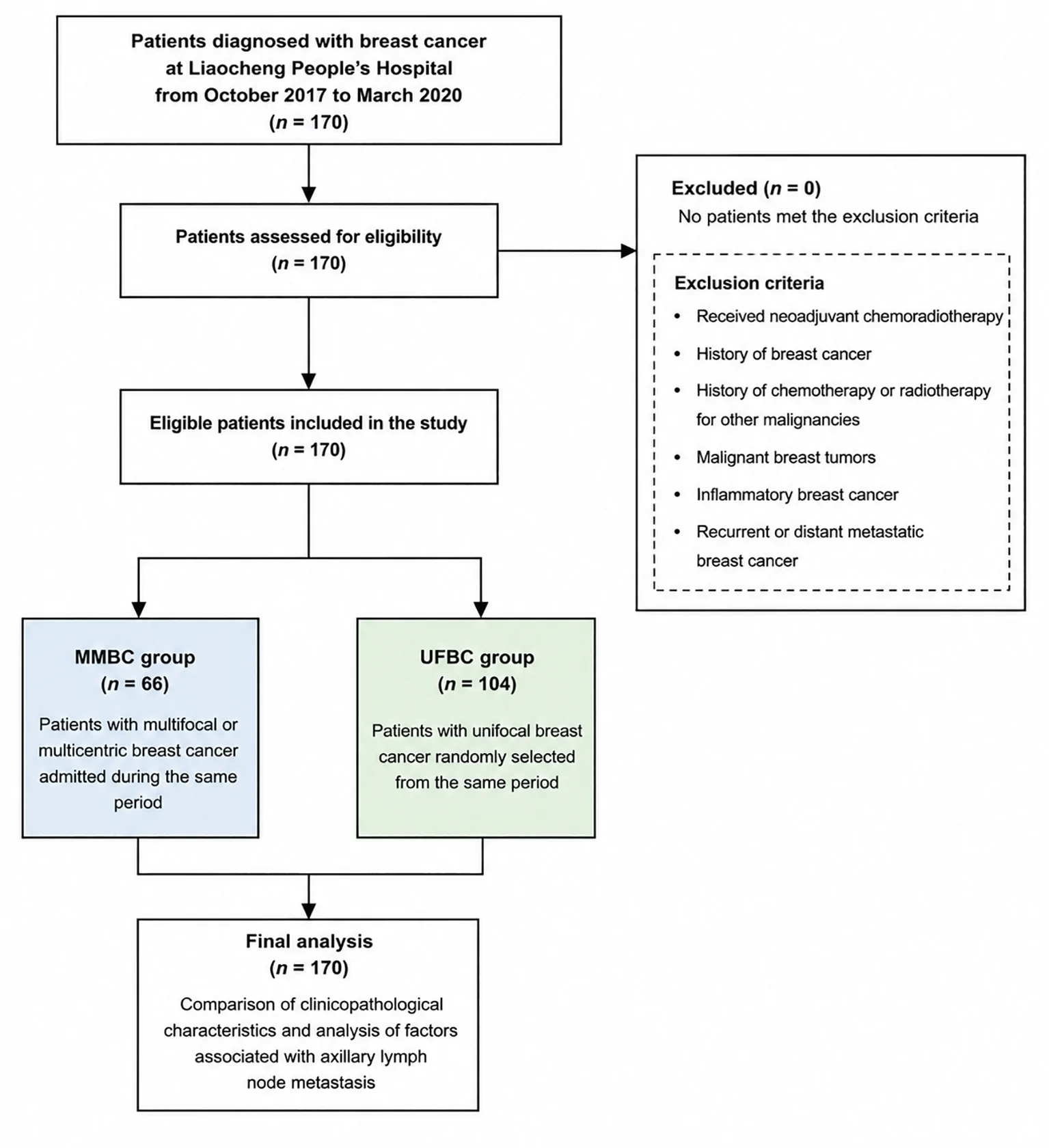
Flowchart of patient screening and study enrollment.

The process of sample collection, exclusion criteria, and subsequent analyses are illustrated in **Figure 1**. From October 2017 to March 2020, 66 patients diagnosed with multifocal and/or multicentric breast cancer were included and analyzed together as the MMBC group at the Department of Breast Surgery, Liaocheng People’s Hospital. During the same study period, 104 patients with UFBC who met the same inclusion and exclusion criteria were randomly selected from the institutional medical-record database as the comparison group. Selection was performed without reference to ALN status or other study outcomes. The UFBC group was not matched to the MMBC group according to tumor size or other clinicopathological characteristics.

Inclusion criteria for MMBC were: (1) postoperative pathological confirmation of malignant breast tumors with lesion distribution consistent with the definition of MMBC; and (2) availability of complete clinical and pathological data. Inclusion criteria for UFBC were: (1) postoperative pathological confirmation of a single malignant breast tumor meeting the definition of UFBC; and (2) availability of complete clinical and pathological data. MMBC was defined as the presence of ≥2 malignant tumors located in different quadrants of the same breast, or ≥2 malignant tumors within the same quadrant separated by normal tissue. Due to the retrospective nature of the study and overlap in imaging and pathological findings, multifocal and multicentric lesions were analyzed together as a single MMBC cohort. UFBC was defined as a single malignant tumor confirmed postoperatively. Data were collected from complete medical records during hospitalization.

Exclusion criteria included: prior neoadjuvant chemoradiotherapy; history of breast cancer; history of chemotherapy or radiotherapy for other malignancies; male breast cancer; inflammatory breast cancer; and recurrent or metastatic breast cancer. The study protocol was reviewed and approved by the Ethics Committee of Liaocheng People’s Hospital (Approval Number: 2025273) and was conducted in accordance with clinical diagnostic and treatment standards. Due to the retrospective nature of the study and the use of anonymized clinical data, the requirement for informed consent was waived by the Ethics Committee of Liaocheng People’s Hospital.

### Collection of pathological indicators

Comprehensive clinical, oncological, and pathological data were collected. The principal variables included patient age, number of tumors, maximum diameter of the largest lesion, total lesion diameter in MMBC patients, T stage, molecular subtype, histological grade, lymphovascular invasion (LVI), presence of intraductal papilloma (IDP), ductal carcinoma *in situ* (DCIS), invasive micropapillary carcinoma (IMPC), and axillary lymph node (ALN) status. LVI was recorded as an overall binary pathological variable. The historical pathological records did not provide sufficiently standardized information to distinguish dermal lymphatic invasion from lymphovascular invasion at other anatomical sites. Pathological skin involvement was not analyzed as a separate predictor. No T4 tumors were present in the study cohort.

### Patient demographics and tumor number

Age was analyzed as an epidemiological factor and categorized as ≤50 years or >50 years for stratified analysis. Tumor number was determined according to the World Health Organization (WHO) histological classification of breast tumors. A single lesion was defined as UFBC, while multiple lesions indicated MMBC. Lesions confined to the same quadrant were classified as multifocal, whereas those located in different quadrants or separated by a significant distance were classified as multicentric. For all analyses in this study, multifocal and multicentric tumors were pooled and analyzed together as a single MMBC cohort, consistent with the study design and previous clinicopathological investigations.

### Tumor size and T stage

The maximum diameter of the largest invasive lesion was used to determine T stage. T stage was classified according to the eighth edition of the American Joint Committee on Cancer (AJCC) TNM staging system as T1 (≤2 cm), T2 (>2 to ≤5 cm), or T3 (>5 cm).21 No T4 tumors were present in the study cohort. For patients with UFBC, total tumor size corresponded to the maximum diameter of the single invasive lesion. For patients with MMBC, total tumor size was calculated as the sum of the maximum diameters of all invasive lesions; the size or extent of any associated *in situ* component, including ductal carcinoma *in situ* (DCIS), was not included. Total tumor size was categorized as ≤2 cm, >2 to ≤5 cm, or >5 cm. In MMBC, the summed diameter of all invasive lesions was used only for the descriptive comparison presented in **Table 1** and was not used for T-stage classification or regression analyses. All T-stage and regression analyses in MMBC were based on the maximum diameter of the largest invasive lesion.

**Table 1:** Comparison of clinicopathological features between MMBC and UFBC.

Variable	Subcategory	UFBC (n = 104)	MMBC (n = 66)	Total (n = 170)	χ²	P-value
Age	≤50	38 (36.54)	35 (53.03)	73 (42.94)	4.482	0.034*
	>50	66 (63.46)	31 (46.97)	97 (57.06)		
T Stage	T1 (≤2 cm)	55 (52.88)	36 (54.55)	91 (53.53)	5.096	0.078
	T2 (>2 to ≤5 cm)	49 (47.12)	27 (40.91)	76 (44.71)		
	T3 (>5 cm)	0 (0.00)	3 (4.55)	3 (1.76)		
Total Tumor Size	T1 (≤2 cm)	55 (52.88)	14 (21.21)	69 (40.59)	34.254	<0.001**
	T2 (>2 to ≤5 cm)	49 (47.12)	37 (56.06)	86 (50.59)		
	T3 (>5 cm)	0 (0.00)	15 (22.73)	15 (8.82)		
Subtype	Luminal A	42 (40.38)	28 (42.42)	70 (41.18)	8.767	0.067
	Luminal B1	27 (25.96)	22 (33.33)	49 (28.82)		
	Luminal B2	14 (13.46)	2 (3.03)	16 (9.41)		
	HER2-enriched	7 (6.73)	9 (13.64)	16 (9.41)		
	Triple-negative	14 (13.46)	5 (7.58)	19 (11.18)		
LVI	No	80 (76.92)	35 (53.03)	115 (67.65)	10.532	0.001**
	Yes	24 (23.08)	31 (46.97)	55 (32.35)		
DCIS	No	50 (48.08)	33 (50.00)	83 (48.82)	0.060	0.807
	Yes	54 (51.92)	33 (50.00)	87 (51.18)		
IDP	No	102 (98.08)	57 (86.36)	159 (93.53)	9.154	0.002**
	Yes	2 (1.92)	9 (13.64)	11 (6.47)		
IMPC	No	94 (90.38)	62 (93.94)	156 (91.76)	0.675	0.411
	Yes	10 (9.62)	4 (6.06)	14 (8.24)		
WHO Grade	I	3 (2.88)	5 (7.58)	8 (4.71)	3.666	0.160
	II	54 (51.92)	39 (59.09)	93 (54.71)		
	III	47 (45.19)	22 (33.33)	69 (40.59)		
Histology (IBC)	IDC	93 (89.42)	57 (86.36)	150 (88.24)	0.364	0.546
	ILC	11 (10.58)	9 (13.64)	20 (11.76)		

UFBC, unifocal breast cancer; MMBC, multifocal/multicentric breast cancer; HER2, human epidermal growth factor receptor 2; LVI, lymphovascular invasion; DCIS, ductal carcinoma in situ; IDP, intraductal papilloma; IMPC, invasive micropapillary carcinoma; WHO, World Health Organization; IBC, invasive breast carcinoma; IDC, invasive ductal carcinoma; ILC, invasive lobular carcinoma. *P < 0.05; **P < 0.01.

DCIS and IDP were recorded as separate, non-mutually exclusive pathological variables.

### Histological grade and pathological features

Histological grading was performed using the modified Bloom–Richardson system (Nottingham Grading System, NGS), which evaluates tubular formation, nuclear pleomorphism, and mitotic activity. Scores of 3–5, 6–7, and 8–9 were classified as low grade (G1), intermediate grade (G2), and high grade (G3), respectively. LVI was assessed using hematoxylin and eosin staining combined with immunohistochemistry, with its presence indicating increased invasiveness and a higher risk of lymph node metastasis. Intraductal papilloma (IDP) was diagnosed as a benign papillary proliferation within a dilated duct, characterized by fibrovascular cores lined by epithelial and myoepithelial cells. DCIS was defined as a malignant epithelial proliferation confined within the mammary ductal-lobular system without invasion through the basement membrane. IDP and DCIS were recorded as separate, non-mutually exclusive pathological variables. IMPC was diagnosed based on characteristic micropapillary tumor-cell clusters with an inside-out growth pattern and was analyzed as an invasive histological feature.

### Molecular biomarkers and detection methods

Molecular subtyping was based on WHO guidelines and assessed using Estrogen receptor (ER), Progesterone receptor (PR), HER2, and Ki-67 expression. ER and PR were evaluated by immunohistochemistry (IHC), with ≥1% nuclear staining defined as positive. HER2 status was assessed by IHC and interpreted according to the current American Society of Clinical Oncology/College of American Pathologists (ASCO/CAP) guidelines. IHC scores of 0 and 1+ were considered negative, 3+ was considered positive, and cases with an IHC score of 2+ were classified as equivocal and underwent fluorescence *in situ* hybridization (FISH) for assessment of HER2 gene amplification (21). Ki-67 expression was assessed as the percentage of positively stained nuclei, with ≤14% defined as low proliferation and >14% as high proliferation. In our institution, ER, PR, HER2, and Ki-67 were routinely assessed in the dominant lesion according to the standard workflow of the pathology department. Biomarker assessment was not routinely performed in all tumor foci.

### Molecular subtyping criteria

Molecular subtypes were classified according to the 2011 St. Gallen surrogate definitions, which were commonly applied during the study period (2017-2020). A Ki-67 cutoff value of 14% was used for surrogate subtype classification in accordance with the institutional practice at that time. We acknowledge that more recent consensus guidelines have updated recommendations regarding Ki-67 interpretation and surrogate molecular classification (6, 7). Luminal A (LA): ER and/or PR positive, HER2 negative, Ki-67 ≤14%; Luminal B1 (LB1): ER and/or PR positive, HER2 negative, Ki-67 >14%; Luminal B2 (LB2): ER and/or PR positive, HER2 positive; HER2-enriched: ER and PR negative, HER2 positive; triple-negative breast cancer (TNBC): negative for ER, PR, and HER2.

### Sentinel and axillary lymph node positivity

Sentinel lymph node (SLN) positivity and axillary lymph node (ALN) positivity were recorded as binary variables (positive or negative). SLN positivity was defined as the presence of metastatic tumor cells in the sentinel lymph nodes, whereas ALN positivity was defined as metastatic involvement of the axillary lymph nodes. The numbers and proportions of patients with SLN positivity and ALN positivity were calculated separately for the UFBC and MMBC groups. Nodal metastasis was available only as a binary positive/negative variable. The historical dataset did not contain sufficiently complete and standardized measurements of metastatic deposit size to classify nodal involvement reliably as isolated tumor cells, micrometastasis, or macrometastasis.

### Statistical methods

Categorical variables were presented as frequencies and percentages. The distributions of age group, T stage, total tumor size, molecular subtype, lymphovascular invasion (LVI), ductal carcinoma *in situ* (DCIS), intraductal papillary (IDP), invasive micropapillary carcinoma (IMPC), WHO histological grade, and histological type were compared between the MMBC and UFBC groups using Pearson’s chi-square test without Yates’ continuity correction. The numbers and proportions of patients with sentinel lymph node (SLN) positivity and axillary lymph node (ALN) positivity were also compared between the MMBC and UFBC groups using Pearson’s chi-square test without Yates’ continuity correction. Positivity rates were calculated using the total number of patients in each group as the denominator: 104 patients in the UFBC group and 66 patients in the MMBC group.

In the univariate analyses of factors associated with ALN metastasis within the UFBC and MMBC groups, Pearson’s chi-square test without Yates’ continuity correction was used when all expected cell counts were ≥5, whereas Fisher’s exact test was used when any expected cell count was <5. Variables with a two-sided P value <0.05 in the univariate analyses were considered candidates for multivariable modeling.

Because of sparse data in some predictor categories and the potential for quasi-complete separation, particularly because all three MMBC patients with T3 tumors had ALN metastasis, Firth-type mean bias-reduced binary logistic regression was used instead of conventional maximum-likelihood logistic regression. ALN status was coded as 0 for negative and 1 for positive.

In the UFBC group, T stage, LVI, and IMPC met the univariate screening criterion and were entered simultaneously into the multivariable model. T stage was treated as a categorical variable, with T1 as the reference category and T2 as the comparison category. LVI and IMPC were treated as binary categorical variables, with their absence specified as the respective reference categories.

In the MMBC group, T stage and LVI met the univariate screening criterion and were entered simultaneously into the multivariable model. T stage was treated as an ordinal numeric variable coded as T1 = 1, T2 = 2, and T3 = 3. Accordingly, its adjusted odds ratio (OR) represented the change in the odds of ALN metastasis associated with each one-category increase in T stage. LVI was treated as a binary categorical variable, with its absence specified as the reference category.

Multicollinearity was assessed using the variance inflation factor (VIF), with a VIF ≥5 considered indicative of potentially important multicollinearity. For both models, regression coefficients, standard errors, Wald χ² statistics, adjusted ORs, and corresponding Wald 95% confidence intervals (CIs) were calculated.

Adjusted ORs and corresponding 95% CIs from the Firth-type bias-reduced logistic regression models were displayed in forest plots. Graphical nomograms were constructed from the coefficients of the corresponding models. Predictor points were assigned in proportion to their modeled regression effects, with the largest modeled effect assigned 100 points. Total points were converted into predicted probabilities of ALN metastasis using the corresponding logistic regression equations. The UFBC nomogram included T stage, LVI, and IMPC. In the MMBC nomogram, T stage was represented as an ordinal predictor, with T1, T2, and T3 assigned progressively increasing point values.

Model performance was evaluated in terms of discrimination, overall predictive accuracy, and calibration. Discrimination was quantified using the area under the receiver operating characteristic curve (AUC). Bootstrap percentile 95% confidence intervals for the apparent AUCs were calculated using 2,000 stratified bootstrap resamples. Overall predictive accuracy was assessed using the Brier score, with lower values indicating greater agreement between the predicted probabilities and observed outcomes. Calibration was examined graphically by comparing the mean predicted probability with the observed proportion of ALN metastasis across groups defined by the ranked predicted probabilities.

Internal validation was performed using 1,000 bootstrap resamples. In each bootstrap sample, the corresponding Firth-type bias-reduced logistic regression model was refitted and its performance was evaluated in both the bootstrap sample and the original dataset. Optimism was estimated as the mean difference between performance in the bootstrap samples and performance in the original dataset. Optimism-corrected AUCs and Brier scores were then obtained by subtracting the estimated optimism from the apparent performance.

Multifocal and multicentric breast cancers were analyzed together as a combined MMBC cohort throughout the study. All statistical tests were two-sided, and P <0.05 was considered statistically significant.

## Results

### Comparison of clinicopathological features between MMBC and UFBC

The proportion of patients aged ≤50 years was significantly higher in the MMBC group than in the UFBC group (53.03% vs. 36.54%; χ² = 4.482, P = 0.034). The distribution of T stage did not differ significantly between the two groups (χ² = 5.096, P = 0.078). In contrast, the distribution of total tumor size differed significantly between MMBC and UFBC (χ² = 34.254, P < 0.001). The proportion of patients with LVI was significantly higher in MMBC than in UFBC (46.97% vs. 23.08%; χ² = 10.532, P = 0.001). Coexisting IDP was significantly more frequent in MMBC than in UFBC (13.64% vs. 1.92%; χ² = 9.154, P = 0.002). No statistically significant differences were observed between the two groups in molecular subtype (P = 0.067), DCIS (P = 0.807), IMPC (P = 0.411), WHO histological grade (P = 0.160), or histological type (P = 0.546) (**Table 1**). In MMBC, the summed diameter of all invasive lesions was used to describe overall tumor burden, whereas T stage was determined using the maximum diameter of the largest invasive lesion.

### Comparison of sentinel and axillary lymph node positivity between MMBC and UFBC

SLN positivity was observed in 25 of 104 patients with UFBC (24.0%) and 14 of 66 patients with MMBC (21.2%). There was no statistically significant difference in SLN positivity between the two groups (χ² = 0.182, P = 0.669). In contrast, ALN positivity was observed in 21 of 104 patients with UFBC (20.2%) and 27 of 66 patients with MMBC (40.9%). The proportion of patients with ALN positivity was significantly higher in the MMBC group than in the UFBC group (χ² = 8.552, P = 0.003) (**Table 2**). This analysis compared nodal positivity rates only and did not assess the diagnostic performance of sentinel lymph node biopsy.

**Table 2:** Comparison of sentinel and axillary lymph node positivity between MMBC and UFBC.

Variable	Group	Positive cases	Positive rate (%)	Total cases	χ²	P-value
SLN+	UFBC	25	24.0	104	0.182	0.669
	MMBC	14	21.2	66		
	Total	39	22.9	170		
ALN+	UFBC	21	20.2	104	8.552	0.003**
	MMBC	27	40.9	66		
	Total	48	28.2	170		

This analysis reports nodal positivity rates only and does not evaluate the diagnostic performance of sentinel lymph node biopsy.

SLN+, sentinel lymph node positivity; ALN+, axillary lymph node metastasis; UFBC, unifocal breast cancer; MMBC, multifocal/multicentric breast cancer. *P < 0.05; **P < 0.01.

### Factors associated with axillary lymph node metastasis in UFBC

In patients with UFBC, univariate analysis showed that ALN metastasis was significantly associated with T stage, LVI, and IMPC (**Table 3**). The ALN metastasis rate was significantly higher in patients with T2 tumors than in those with T1 tumors (30.61% vs. 10.91%; χ² = 6.243, P = 0.012). Patients with LVI had a significantly higher ALN metastasis rate than those without LVI (41.67% vs. 13.75%; Fisher’s exact P = 0.007). Similarly, patients with IMPC had a significantly higher ALN metastasis rate than those without IMPC (50.00% vs. 17.02%; Fisher’s exact P = 0.027). IDP was not significantly associated with ALN metastasis in UFBC. In patients with UFBC, univariate analysis showed that ALN metastasis was significantly associated with T stage, LVI, and IMPC (**Table 3**). The ALN metastasis rate was significantly higher in patients with T2 tumors than in those with T1 tumors (30.61% vs. 10.91%; χ² = 6.243, P = 0.012). Patients with LVI had a significantly higher ALN metastasis rate than those without LVI (41.67% vs. 13.75%; Fisher’s exact P = 0.007). Similarly, patients with IMPC had a significantly higher ALN metastasis rate than those without IMPC (50.00% vs. 17.02%; Fisher’s exact P = 0.027). IDP was not significantly associated with ALN metastasis. Thus, the micropapillary feature associated with ALN metastasis was IMPC, an invasive carcinoma pattern, rather than IDP, a benign intraductal papillary lesion. No statistically significant associations with ALN metastasis were observed for age group, molecular subtype, DCIS, IDP, WHO histological grade, or histological type (all P >0.05).

**Table 3:** Factors associated with axillary lymph node metastasis in UFBC.

Variable	Category	ALN+ cases	Total cases	Metastasis rate (%)	Test	χ²	P-value
Age	≤50 years	6	38	15.79	Pearson chi-square	0.720	0.396
	>50 years	15	66	22.73			
T Stage	T1 (≤2 cm)	6	55	10.91	Pearson chi-square	6.243	0.012*
	T2 (>2 to ≤5 cm)	15	49	30.61			
Subtype	Luminal A	9	42	21.43	Fisher’s exact test	–	0.218
	Luminal B1	8	27	29.63			
	Luminal B2	3	14	21.43			
	HER2-enriched	1	7	14.29			
	Triple-negative	0	14	0.00			
LVI	No	11	80	13.75	Fisher’s exact test	–	0.007**
	Yes	10	24	41.67			
DCIS	No	13	50	26.00	Pearson chi-square	2.016	0.156
	Yes	8	54	14.81			
IDP	No	21	102	20.59	Fisher’s exact test	–	1.000
	Yes	0	2	0.00			
IMPC	No	16	94	17.02	Fisher’s exact test	–	0.027*
	Yes	5	10	50.00			
WHO Grade	I	1	3	33.33	Fisher’s exact test	–	0.512
	II	12	54	22.22			
	III	8	47	17.02			
Histology (IBC)	IDC	18	93	19.35	Fisher’s exact test	–	0.691
	ILC	3	11	27.27			

ALN+, axillary lymph node metastasis; UFBC, unifocal breast cancer; HER2, human epidermal growth factor receptor 2; LVI, lymphovascular invasion; DCIS, ductal carcinoma in situ; IDP, intraductal papillary carcinoma; IMPC, invasive micropapillary carcinoma; WHO, World Health Organization; IBC, invasive breast carcinoma; IDC, invasive ductal carcinoma; ILC, invasive lobular carcinoma.

Pearson’s chi-square test without Yates’ continuity correction was used when all expected cell counts were ≥5. Fisher’s exact test was used when any expected cell count was <5. A dash indicates that the χ² statistic is not applicable to Fisher’s exact test.

*P < 0.05; **P < 0.01.

T stage, LVI, and IMPC met the univariate screening criterion and were included in the UFBC Firth-type bias-reduced logistic regression model. No important multicollinearity was detected among the predictors; the VIF values were 1.002 for T stage, 1.003 for LVI, and 1.005 for IMPC.

**Figure 2:**
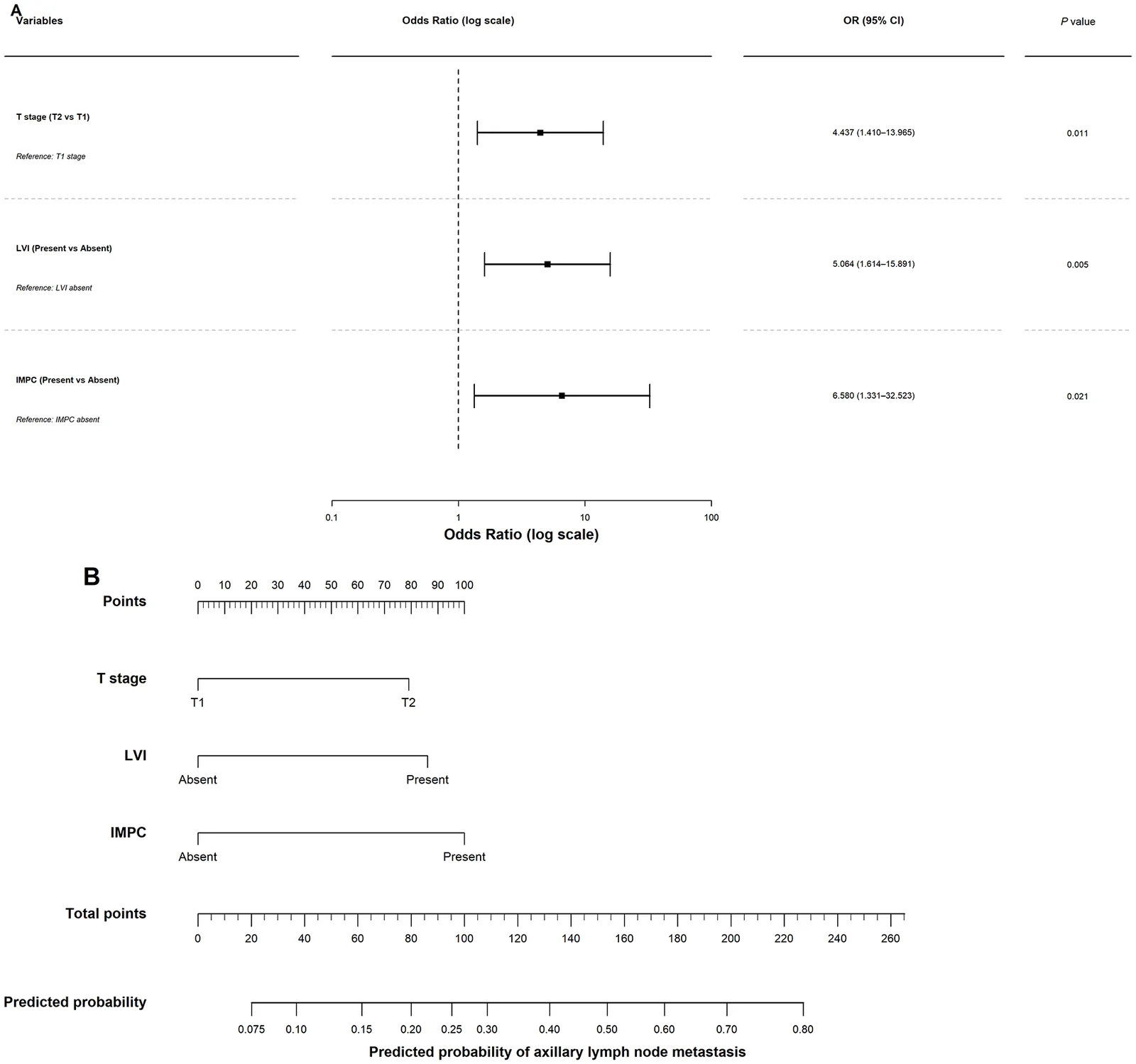
Firth-type bias-reduced logistic regression analysis and nomogram for axillary lymph node metastasis in UFBC. **(A)** Forest plot showing adjusted odds ratios (ORs) and 95% confidence intervals (CIs) for T stage, lymphovascular invasion (LVI), and invasive micropapillary carcinoma (IMPC). T1 stage, absence of LVI, and absence of IMPC were used as the reference categories. **(B)** Nomogram constructed from the same model to estimate the predicted probability of axillary lymph node metastasis based on T stage, LVI, and IMPC. Higher total points correspond to a higher predicted probability.

After mutual adjustment, all three predictors remained significantly associated with ALN metastasis (**Table 4**; **Figure 2A**). Compared with T1 tumors, T2 tumors were associated with significantly higher odds of ALN metastasis (B = 1.490, adjusted OR = 4.437, 95% CI: 1.410-13.965, P = 0.011). The presence of LVI was associated with significantly higher odds of ALN metastasis compared with the absence of LVI (B = 1.622, adjusted OR = 5.064, 95% CI: 1.614-15.891, P = 0.005). The presence of IMPC was also associated with significantly higher odds of ALN metastasis compared with the absence of IMPC (B = 1.884, adjusted OR = 6.580, 95% CI: 1.331-32.523, P = 0.021).

**Table 4:** Firth-type bias-reduced logistic regression analysis of factors associated with axillary lymph node metastasis in UFBC.

Variable	Coefficient (B)	Standard error	Wald χ²	df	P-value	Adjusted OR	95% CI for Adjusted OR
T stage: T2 vs. T1	1.490	0.585	6.488	1	0.011	4.437	1.410-13.965
LVI: present vs. absent	1.622	0.583	7.730	1	0.005	5.064	1.614-15.891
IMPC: present vs. absent	1.884	0.815	5.341	1	0.021	6.580	1.331-32.523
Intercept	−2.893	0.562	26.512	1	<0.001	0.055	0.018-0.167

ALN positivity was coded as 0 for negative and 1 for positive. The model was fitted using Firth-type mean bias-reduced logistic regression. T1 stage, absence of LVI, and absence of IMPC were used as the reference categories. Adjusted ORs and 95% CIs were derived from the multivariable model. Results should be interpreted cautiously because of the limited numbers of patients in some categories.

UFBC, unifocal breast cancer; ALN, axillary lymph node; LVI, lymphovascular invasion; IMPC, invasive micropapillary carcinoma; OR, odds ratio; CI, confidence interval.

A nomogram incorporating T stage, LVI, and IMPC was constructed from the same Firth-type bias-reduced logistic regression model to estimate the predicted probability of ALN metastasis (**Figure 2B**). Higher total points corresponded to a higher predicted probability. The model and nomogram require further validation before clinical application.

### Factors associated with axillary lymph node metastasis in MMBC

In patients with MMBC, univariate analysis showed that ALN metastasis was significantly associated with the T stage of the largest invasive lesion and the presence of LVI (**Table 5**). The ALN metastasis rates were 30.56% for T1 tumors, 48.15% for T2 tumors, and 100.00% for T3 tumors, with a statistically significant difference across T-stage categories (Fisher’s exact P = 0.035). Patients with LVI had a significantly higher ALN metastasis rate than those without LVI (54.84% vs. 28.57%; χ² = 4.692, P = 0.030). No statistically significant associations with ALN metastasis were observed for age group, total tumor diameter, molecular subtype, DCIS, IDP, IMPC, WHO histological grade, or histological type (all P >0.05).

**Table 5:** Factors associated with axillary lymph node metastasis in MMBC.

Variable	Category	ALN+ cases	Total cases	Metastasis rate (%)	Test	χ²	P-value
Age	≤50 years	15	35	42.86	Pearson chi-square	0.117	0.732
	>50 years	12	31	38.71			
T Stage	T1 (≤2 cm)	11	36	30.56	Fisher’s exact test	–	0.035*
	T2 (>2 to ≤5 cm)	13	27	48.15			
	T3 (>5 cm)	3	3	100.00			
Total Tumor Diameter	≤2 cm	4	14	28.57	Pearson chi-square	3.287	0.193
	>2 to ≤5 cm	14	37	37.84			
	>5 cm	9	15	60.00			
Subtype	Luminal A	9	28	32.14	Fisher’s exact test	–	0.075
	Luminal B1	14	22	63.64			
	Luminal B2	0	2	0.00			
	HER2-enriched	2	9	22.22			
	Triple-negative	2	5	40.00			
LVI	No	10	35	28.57	Pearson chi-square	4.692	0.030*
	Yes	17	31	54.84			
DCIS	No	14	33	42.42	Pearson chi-square	0.063	0.802
	Yes	13	33	39.39			
IDP	No	24	57	42.11	Fisher’s exact test	–	0.727
	Yes	3	9	33.33			
IMPC	No	24	62	38.71	Fisher’s exact test	–	0.297
	Yes	3	4	75.00			
WHO Grade	I	2	5	40.00	Fisher’s exact test	–	0.281
	II	19	39	48.72			
	III	6	22	27.27			
Histology (IBC)	IDC	23	57	40.35	Fisher’s exact test	–	1.000
	ILC	4	9	44.44			

T stage was determined according to the diameter of the largest invasive lesion in accordance with the AJCC staging criteria.

MMBC, multifocal/multicentric breast cancer; ALN+, axillary lymph node metastasis; HER2, human epidermal growth factor receptor 2; LVI, lymphovascular invasion; DCIS, ductal carcinoma in situ; IDP, intraductal papillary carcinoma; IMPC, invasive micropapillary carcinoma; WHO, World Health Organization; IBC, invasive breast carcinoma; IDC, invasive ductal carcinoma; ILC, invasive lobular carcinoma.

Pearson’s chi-square test without Yates’ continuity correction was used when all expected cell counts were ≥5. Fisher’s exact test was used when any expected cell count was <5. A dash indicates that the χ² statistic is not applicable to Fisher’s exact test.

*P < 0

Ordinal T stage and LVI met the univariate screening criterion and were included in the MMBC Firth-type bias-reduced logistic regression model. No important multicollinearity was detected between the predictors, with a VIF of 1.089 for both ordinal T stage and LVI.

**Figure 3:**
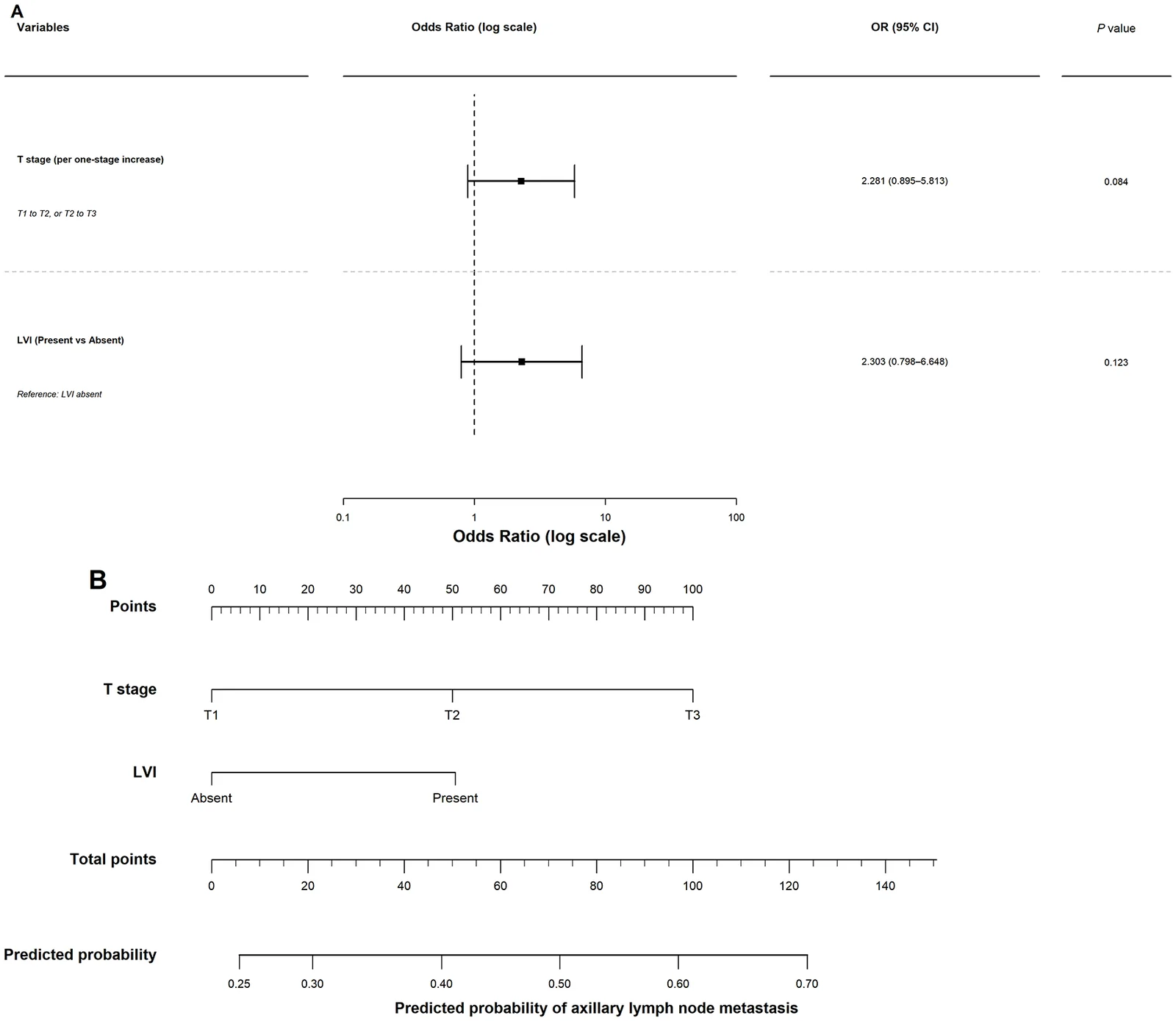
Firth-type bias-reduced logistic regression analysis and nomogram for axillary lymph node metastasis in MMBC. **(A)** Forest plot showing adjusted odds ratios (ORs) and 95% confidence intervals (CIs) for ordinal T stage and lymphovascular invasion (LVI). T stage was modeled as an ordinal variable coded as T1 = 1, T2 = 2, and T3 = 3; therefore, its adjusted OR represents the change in the odds of axillary lymph node metastasis associated with each one-category increase from T1 to T2 or from T2 to T3. Absence of LVI was used as the reference category. **(B)** Exploratory nomogram constructed from the same model to estimate the predicted probability of axillary lymph node metastasis based on ordinal T stage and LVI. Higher total points correspond to a higher predicted probability.

After mutual adjustment, neither ordinal T stage nor LVI reached statistical significance (**Table 6**; **Figure 3A**). Each one-category increase in T stage was associated with an estimated 2.281-fold increase in the odds of ALN metastasis, although this association was not statistically significant (B = 0.825, adjusted OR = 2.281, 95% CI: 0.895-5.813, P = 0.084). The presence of LVI was associated with an estimated 2.303-fold increase in the odds of ALN metastasis compared with the absence of LVI, but this association was also not statistically significant (B = 0.834, adjusted OR = 2.303, 95% CI: 0.798-6.648, P = 0.123).

**Table 6:** Firth-type bias-reduced logistic regression analysis of factors associated with axillary lymph node metastasis in MMBC.

Variable	Coefficient (B)	Standard error	Wald χ²	df	P-value	Adjusted OR	95% CI for adjusted OR
T stage: per one-category increase	0.825	0.477	2.983	1	0.084	2.281	0.895-5.813
LVI: present vs. absent	0.834	0.541	2.381	1	0.123	2.303	0.798-6.648
Intercept	−2.018	0.773	6.805	1	0.009	0.133	0.029-0.605

ALN positivity was coded as 0 for negative and 1 for positive. The model was fitted using Firth-type mean bias-reduced logistic regression. T stage was determined from the largest invasive lesion according to the AJCC staging criteria and was entered as an ordinal variable coded as T1 = 1, T2 = 2, and T3 = 3. Accordingly, the adjusted OR for T stage represents the change in the odds of ALN metastasis associated with each one-category increase. Absence of LVI was used as the reference category. Results should be interpreted cautiously because of the limited sample size and the small number of patients in some T-stage categories.

MMBC, multifocal/multicentric breast cancer; ALN, axillary lymph node; LVI, lymphovascular invasion; AJCC, American Joint Committee on Cancer; OR, odds ratio; CI, confidence interval.

An exploratory nomogram incorporating ordinal T stage and LVI was constructed from the same Firth-type bias-reduced logistic regression model (**Figure 3B**). Higher total points corresponded to a higher predicted probability of ALN metastasis. These findings should be interpreted cautiously because of the limited sample size and the small number of patients in some T-stage categories, and the model requires further validation.

Overall, the factors associated with ALN metastasis differed between the UFBC and MMBC groups. In the UFBC group, T stage, LVI, and IMPC remained statistically significant in the Firth-type multivariable model. In the MMBC group, T stage and LVI were significantly associated with ALN metastasis in the univariate analyses; however, neither predictor reached statistical significance after mutual adjustment in the Firth-type multivariable model. In all MMBC analyses, T stage was determined according to the maximum diameter of the largest invasive lesion rather than the summed diameter of all lesions.

### Nomogram performance and internal validation

The UFBC nomogram demonstrated moderate apparent discrimination, with an AUC of 0.773 (95% CI: 0.665-0.863). After internal validation, the optimism-corrected AUC was 0.746. The apparent Brier score was 0.128, and the optimism-corrected Brier score was 0.142. Graphical assessment of calibration is presented in **Supplementary Figure 1A**.

The MMBC nomogram showed lower apparent discrimination, with an AUC of 0.688 (95% CI: 0.563-0.810). The optimism-corrected AUC was 0.664. The apparent and optimism-corrected Brier scores were 0.213 and 0.232, respectively. Graphical assessment of calibration is presented in **Supplementary Figure 1B**.

The internal-validation results are summarized in **Table 7**. Overall, the UFBC model retained moderate discrimination after optimism correction, whereas the MMBC model showed more limited predictive performance. Both models remain exploratory and require external validation before clinical application.

**Table 7:** Performance and bootstrap internal validation of the UFBC and MMBC nomogram models.

Model	Apparent AUC (95% CI)	Optimism-corrected AUC	Apparent Brier score	Optimism-corrected Brier score	Successful bootstrap resamples
UFBC	0.773 (0.665-0.863)	0.746	0.128	0.142	999
MMBC	0.688 (0.563-0.810)	0.664	0.213	0.232	1,000

Internal validation was performed using 1,000 bootstrap resamples. Higher AUC values indicate better discrimination, whereas lower Brier scores indicate better overall predictive accuracy. One UFBC bootstrap iteration did not yield a valid model fit; therefore, 999 successful resamples were used for its optimism correction.

UFBC, unifocal breast cancer; MMBC, multifocal/multicentric breast cancer; AUC, area under the receiver operating characteristic curve; CI, confidence interval.

## Discussion

The pathogenesis and biological organization of multifocal and multicentric breast cancer remain incompletely understood. Previous studies have proposed that multiple ipsilateral tumor foci may arise either independently or through intramammary dissemination from a primary lesion (22). Molecular and clonality studies have also demonstrated heterogeneity among individual tumor foci. However, these mechanisms were not directly evaluated in the present retrospective study. Because multifocal and multicentric lesions could not always be reliably distinguished in the available imaging and pathological records, they were analyzed together as a combined MMBC cohort. This grouping was adopted for practical and analytical reasons and should not be interpreted as evidence that multifocal and multicentric tumors are biologically identical.

In this cohort, patients aged ≤50 years accounted for a significantly greater proportion of the MMBC group than of the UFBC group. This finding suggests that MMBC may have a different age distribution from UFBC in the present population. Nutter et al. (23) reported an association between younger age, hormonal sensitivity, and increased multifocality. Nevertheless, menopausal status and other reproductive or hormonal factors were not available in our dataset. Therefore, the observed age difference cannot be directly attributed to hormonal mechanisms and requires confirmation in larger studies with more comprehensive clinical information.

LVI was significantly more frequent in MMBC than in UFBC. LVI represents tumor-cell involvement of lymphatic or vascular spaces and has been associated with lymphatic dissemination, recurrence, and adverse outcomes in breast cancer. Yoon et al. (24) similarly reported an association between LVI and multifocal disease, local recurrence, and distant metastasis. The higher frequency of LVI observed in the MMBC group may therefore reflect more frequent lymphovascular involvement in patients with multiple tumor foci. However, the cross-sectional and retrospective nature of the present analysis does not establish a causal relationship between multifocality or multicentricity and LVI.

Coexisting IDP was significantly more frequent in MMBC than in UFBC, whereas the prevalence of DCIS did not differ significantly between the groups. IDP and DCIS were analyzed as separate pathological variables because IDP represents a benign papillary proliferation, whereas DCIS represents a noninvasive malignant epithelial proliferation. These variables were not considered mutually exclusive because both lesions could coexist in the same pathological specimen. Importantly, neither IDP nor DCIS was significantly associated with ALN metastasis within the UFBC or MMBC group. Therefore, the higher frequency of IDP in MMBC should not be interpreted as evidence that IDP contributed to the higher ALN positivity rate observed in MMBC. This finding should be interpreted cautiously and requires confirmation in a larger cohort with centralized pathological review.

Molecular subtype distribution did not differ significantly between MMBC and UFBC. Although Luminal B1 and HER2-enriched tumors were numerically more frequent in the MMBC group, these differences did not reach statistical significance and should not be interpreted as evidence of subtype enrichment. Previous studies have reported variable molecular-subtype distributions in MMBC, including increased proportions of Luminal B or HER2-positive disease in some populations (25). Differences among studies may reflect variations in cohort composition, biomarker assessment, subtype definitions, and inter-lesional heterogeneity. In the present study, biomarkers were routinely assessed in the dominant lesion rather than in every tumor focus. In addition, molecular subtyping was based on the 2011 St. Gallen surrogate definitions and a Ki-67 cutoff of 14%, consistent with institutional practice during 2017-2020. More recent consensus recommendations have revised the interpretation of Ki-67 and surrogate molecular classification (6, 7). Accordingly, the subtype results should be interpreted within their historical methodological context.

WHO histological grade and histological type did not differ significantly between MMBC and UFBC. Although previous studies have associated higher histological grade with adverse outcomes (26), the present findings do not demonstrate a significant difference in grade distribution between the two groups. ILC was numerically more frequent in MMBC, consistent with its known tendency toward multifocal, multicentric, and bilateral growth arising from its discohesive histological pattern (27), but the difference was not statistically significant. The limited number of patients with ILC may have reduced the ability to detect a group difference.

ALN positivity was significantly more frequent in MMBC than in UFBC, whereas SLN positivity did not differ significantly between the groups. The clinical relevance of these findings should also be considered in the context of ongoing de-escalation of axillary surgery. The SOUND randomized clinical trial demonstrated that omission of axillary surgery was noninferior to SLNB with respect to 5-year distant disease-free survival in carefully selected patients with small invasive breast cancers and negative preoperative axillary ultrasonography. However, the SOUND trial was restricted to patients with unifocal tumors and therefore did not establish the safety of omitting SLNB in patients with MMBC. This distinction is clinically important because MMBC was associated with a significantly higher ALN positivity rate than UFBC in our cohort. Consequently, the SOUND findings should not be directly extrapolated to patients with MMBC, and dedicated prospective evidence is required before omission of SLNB can be considered in this population. These results indicate different observed patterns of nodal involvement in this cohort but do not evaluate the diagnostic accuracy of sentinel lymph node biopsy. The study did not assess sensitivity, specificity, false-negative rates, or long-term outcomes associated with sentinel lymph node biopsy. Although contemporary evidence supports sentinel lymph node biopsy as a feasible staging procedure in appropriately selected patients with MMBC (28), the present findings should not be interpreted as supporting one axillary staging or surgical strategy over another.

The method used to determine tumor size in MMBC remains an important issue. Current AJCC staging is based on the largest invasive focus rather than the sum of multiple lesion diameters. However, reliance on the largest focus alone may underestimate the overall tumor burden in patients with multiple lesions (20). Conversely, aggregate tumor size has not been standardized for clinical staging and remains insufficiently validated for treatment planning or prognostic assessment. In this study, T stage was determined from the largest invasive lesion in accordance with AJCC criteria, whereas the summed diameter of all invasive lesions was used only as a descriptive measure of overall tumor burden.

In the MMBC group, T stage based on the largest invasive lesion was significantly associated with ALN metastasis in the univariate analysis. The ALN metastasis rate increased from 30.56% for T1 to 48.15% for T2 and 100.00% for T3. Nevertheless, only three patients had T3 tumors, and all three had ALN metastasis, producing sparse data and potential quasi-complete separation. After adjustment for LVI using Firth-type bias-reduced logistic regression, each one-category increase in T stage was associated with an adjusted OR of 2.281, but the association did not reach statistical significance. LVI was also significant in the univariate analysis but not in the adjusted model. Therefore, neither ordinal T stage nor LVI can be considered independently associated with ALN metastasis in MMBC on the basis of the present multivariable results. The observed effect estimates may nevertheless warrant evaluation in larger cohorts.

In the UFBC group, T stage, LVI, and IMPC remained significantly associated with ALN metastasis in the Firth-type multivariable model. Compared with T1 tumors, T2 tumors were associated with higher odds of ALN metastasis. The presence of LVI was also associated with increased odds of ALN metastasis, consistent with its biological role in lymphatic dissemination. IMPC showed the largest adjusted OR among the included UFBC predictors. IMPC is a relatively uncommon but aggressive histological subtype characterized by a high propensity for lymphovascular invasion and lymph node involvement (29). However, only 10 UFBC patients had IMPC, and the corresponding confidence interval was wide. The magnitude of this association should therefore be interpreted cautiously and validated in a larger sample.

Firth-type mean bias-reduced logistic regression was used because several categories contained small numbers of patients and the MMBC T3 category exhibited potential quasi-complete separation (30–32). This approach reduced the risk of infinite or markedly biased estimates associated with conventional maximum-likelihood logistic regression. The low VIF values indicated no important multicollinearity among the predictors included in either model. Nevertheless, bias-reduced estimation cannot fully compensate for limited sample size, sparse predictor categories, or the restricted number of outcome events.

The UFBC nomogram incorporating T stage, LVI, and IMPC demonstrated an apparent AUC of 0.773 and an optimism-corrected AUC of 0.746, indicating moderate discrimination after internal validation. Its apparent and optimism-corrected Brier scores were 0.128 and 0.142, respectively. In comparison, the MMBC nomogram incorporating ordinal T stage and LVI showed more limited discrimination, with an apparent AUC of 0.688 and an optimism-corrected AUC of 0.664. Its apparent and optimism-corrected Brier scores were 0.213 and 0.232, respectively. Graphical calibration assessments are provided in **Supplementary Figure 1**. These findings suggest that the UFBC model retained moderate predictive ability after correction for optimism, whereas the MMBC model had comparatively limited performance. Both nomograms should be regarded as exploratory visual representations of the fitted models rather than clinically validated decision tools.

Overall, MMBC was associated with a higher observed ALN positivity rate and more frequent LVI and IDP than UFBC in this cohort. Within UFBC, T stage, LVI, and IMPC remained significantly associated with ALN metastasis after multivariable adjustment. Within MMBC, T stage and LVI were associated with ALN metastasis in the univariate analyses, but neither reached statistical significance after mutual adjustment. These findings underscore the need for larger multicenter studies to clarify whether clinicopathological factors have different associations with nodal involvement in MMBC and UFBC.

### Limitations of the study

This study has several limitations. First, it was a single-center retrospective study with a relatively small sample size, particularly in the MMBC group. Several clinicopathological categories contained few patients; notably, only three MMBC patients had T3 tumors, and all three had ALN metastasis. Although Firth-type mean bias-reduced logistic regression was used to reduce sparse-data and separation-related bias, the resulting estimates remained imprecise, as reflected by the wide confidence intervals. The absence of statistical significance in the adjusted MMBC model may partly reflect limited statistical power and should not be interpreted as evidence of no association.

Second, the UFBC control group was randomly selected rather than matched to the MMBC group according to baseline clinicopathological characteristics. Differences between the groups may therefore have been influenced by selection bias, residual confounding, or both. Moreover, inclusion in the multivariable models was based on statistical significance in the univariate analyses. This data-dependent selection strategy may result in unstable predictor selection, particularly in a relatively small dataset. The multivariable findings should therefore be interpreted as exploratory associations rather than definitive causal effects.

Third, multifocal and multicentric breast cancers were combined into a single MMBC cohort. This approach was necessary because of overlap in their imaging and pathological classification in the historical records and the insufficient sample sizes available for reliable separate analyses. Nevertheless, multifocal and multicentric tumors may differ in their biological origins, spatial distribution, molecular characteristics, and clinical behavior. Combining them may therefore have obscured clinically relevant heterogeneity between these entities.

Fourth, biomarker assessment was routinely performed in the dominant lesion and was not available for every tumor focus. Inter-lesional heterogeneity in ER, PR, HER2, and Ki-67 expression could therefore not be evaluated. Molecular subtype classification was based on the 2011 St. Gallen surrogate definitions and a fixed Ki-67 cutoff of 14%, reflecting institutional practice during the study period. Because more recent consensus guidelines have revised the interpretation of Ki-67 and surrogate molecular classification,6,7 some tumors might be classified differently under contemporary criteria.

Fifth, T stage in MMBC was determined from the largest invasive lesion in accordance with the AJCC staging system. Although the summed diameter of all invasive lesions was recorded as a descriptive measure of overall tumor burden, the study was not designed to compare the ability of the largest-lesion measurement and aggregate tumor size to characterize the risk of ALN metastasis. It therefore remains uncertain whether summed tumor burden provides additional information beyond conventional T stage. In addition, LVI was available only as an overall binary variable. Dermal lymphatic invasion and pathological skin involvement were not recorded with sufficient consistency to permit separate analyses. Their potential associations with ALN metastasis could therefore not be evaluated.

Sixth, although the UFBC and MMBC nomograms underwent bootstrap internal validation, they were not externally validated. The optimism-corrected AUC was 0.746 for the UFBC model and 0.664 for the MMBC model, with the latter indicating limited discrimination. Calibration was assessed graphically, but the relatively small sample sizes restricted the precision and reliability of the calibration estimates. The nomograms should therefore be regarded as exploratory and should not be used for clinical decision-making until they have been validated in larger independent cohorts.

Seventh, the study did not include follow-up information on local recurrence, distant metastasis, disease-free survival, or overall survival. Consequently, the identified factors can only be interpreted in relation to ALN metastasis and cannot be considered prognostic factors for long-term clinical outcomes. In addition, the study did not assess the diagnostic accuracy, false-negative rate, or clinical outcomes of sentinel lymph node biopsy and was not designed to guide the selection of axillary surgical procedures. ALN status was analyzed only as a binary outcome. The available data did not permit reliable classification of nodal involvement as isolated tumor cells, micrometastasis, or macrometastasis, or reliable distinction between patients with one to two and those with more than two metastatic axillary lymph nodes. Therefore, this study could not determine whether the pathological features of the primary tumor differed according to metastatic deposit size or identify predictors of high nodal burden. Its findings cannot be used to support decisions regarding omission of axillary lymph node dissection.

Future multicenter prospective studies with larger cohorts, standardized contemporary biomarker assessment across all tumor foci, detailed documentation of dermal lymphatic and pathological skin involvement and the numbers of examined and metastatic lymph nodes, external validation of the prediction models, and longitudinal outcome data are required to confirm and extend these findings.

## Conclusions

In this retrospective single-center cohort, MMBC was characterized by higher frequencies of LVI, coexisting IDP and ALN positivity than UFBC. In the UFBC group, T stage, LVI, and IMPC remained significantly associated with ALN metastasis in the Firth-type bias-reduced multivariable analysis. In the MMBC group, T stage based on the largest invasive lesion and LVI were associated with ALN metastasis in the univariate analyses, but neither remained statistically significant after mutual adjustment. The UFBC nomogram demonstrated moderate discrimination after bootstrap internal validation, whereas the MMBC nomogram showed more limited predictive performance. These models should be considered exploratory and require external validation. Larger multicenter prospective studies with contemporary biomarker assessment and longitudinal outcome data are needed to confirm these findings.

## Data Availability

The datasets presented in this study can be found in online repositories. The names of the repository/repositories and accession number(s) can be found in the article/**Supplementary Material**.

## Supplementary material

Supplementary Figure 1 Calibration plots of the nomogram models for predicting axillary lymph node metastasis. **(A)** Calibration plot for the UFBC nomogram incorporating T stage, lymphovascular invasion, and invasive micropapillary carcinoma. **(B)** Calibration plot for the MMBC nomogram incorporating ordinal T stage and lymphovascular invasion. The diagonal dashed line represents perfect agreement between predicted probabilities and observed proportions, and the solid line represents the observed graphical calibration.
